# Potential Use of Atlantic Cod Trypsin in Biomedicine

**DOI:** 10.1155/2013/749078

**Published:** 2013-02-28

**Authors:** Ágústa Gudmundsdóttir, Hilmar Hilmarsson, Bjarki Stefansson

**Affiliations:** ^1^Department of Biochemistry, Science Institute, University of Iceland, Dunhagi 5, 107 Reykjavik, Iceland; ^2^Faculty of Food Science and Nutrition, School of Health Sciences and Science Institute, University of Iceland, Dunhagi 5, 107 Reykjavik, Iceland

## Abstract

Surface proteins of viruses and bacteria used for cell attachment and invasion are candidates for degradation by proteases. Trypsin from Atlantic cod (*Gadus morhua*) was previously demonstrated to have efficacy against influenza viruses *in vitro* and on skin. In this paper, cod trypsin is shown to be 3–12 times more effective in degrading large native proteins than its mesophilic analogue, bovine trypsin. This is in agreement with previous findings where cod trypsin was found to be the most active among twelve different proteases in cleaving various cytokines and pathological proteins. Furthermore, our results show that cod trypsin has high efficacy against herpes simplex virus type 1 (HSV-1) and the respiratory syncytial virus (RSV) *in vitro*. The results on the antipathogenic properties of cod trypsin are important because rhinovirus, RSV, and influenza are the most predominant pathogenic viruses in upper respiratory tract infections. Results from a clinical study presented in this paper show that a specific formulation containing cod trypsin was preferred for wound healing over other methods used in the study. Apparently, the high digestive ability of the cold-adapted cod trypsin towards large native proteins plays a role in its efficacy against pathogens and its positive effects on wounds.

## 1. Introduction

This paper presents basic and clinical research conducted on the cold-adapted Atlantic cod trypsin for the past decade with emphasis on its use in biomedicine. A previous review article, covering 25 years of basic research on Atlantic cod trypsin and its practical applications, has gained high interest by the scientific community since its publication [1]. Research data on protein digestion [2, 3] and *in vitro* efficacy of the enzyme against viruses [4] is introduced as well as clinical data on the use of cod trypsin in wound healing [5].

Atlantic cod trypsin, a cold-adapted serine protease, has higher catalytic efficiency and it is more sensitive to inactivation by heat, low pH, and autolysis than its mesophilic bovine analogue [6–8]. Atlantic cod trypsin and salmon trypsin [9] are typical of the traditionally classified cold-adapted trypsins and have been extensively studied [1, 10]. These 2 trypsins have about 80% amino acid sequence identity [11], and they appear to share a nearly identical 3-dimensional structure, as previously demonstrated by modeling studies [10, 12]. Cod trypsin is purified from the byproducts of Atlantic cod (*Gadus morhua*), making its production both economically and environmentally friendly [1, 13]. For these reasons, cod trypsin has been tested and used for different biomedical applications. Trypsin and chymotrypsin from the pancreas of cattle or pigs have been used for decades as therapeutic agents in clinical trials for humans and animals [14–19]. The recombinant form of Atlantic cod trypsin has been produced in an active form in microorganisms [20, 21].

Previous studies show that cod trypsin and salmon trypsin have higher efficacy on small chromogenic substrates than bovine trypsin [7, 8, 22]. One of the disadvantages of using small synthetic substrates for physiological measurements of protease activity is that they obviously do not fully represent natural proteins. Proteases may cleave small synthetic substrates whereas their access to physiological proteins in their native form can be somewhat hindered [23]. Therefore, it is important to study the catalytic efficacy of cod trypsin towards natural proteins with regard to its biomedical applications. In general, lower enzyme concentrations are preferred in biomedicine for safety as well as for environmental and economical reasons [1]. Cod trypsin in a specific hydrogel formulation was in a clinical trial found to be effective in reducing the volume of pressure sores [5]. This is in agreement with previous reports on wound healing using high concentrations of mammalian trypsins [18]. 

Surface proteins of microbes such as viruses and bacteria are important for cell attachment and invasion of the pathogens into cells [24, 25]. The ability of cod trypsin to efficiently degrade proteins on the surface of pathogens is postulated to be the basis for its antipathogenic efficacy [3, 8]. This has been strongly indicated in several studies. For example, Ahmad et al. [26] showed that cod trypsin can be used to cleave and quantify proteins on the surface of cultured mouse cells in the absence of membrane trafficking. Also, cod trypsin was found to be the most active among 12 comparable proteases including cold-adapted euphaulysin protease from Antarctic krill in cleaving various cytokines and pathological proteins [27]. In addition, cod trypsin has been demonstrated to remove biofilm caused by *Pseudomonas* bacteria in dairy equipment [28]. Berg et al. showed that cold-adapted serine proteases from Antarctic krill can be effective in the enzymatic removal of dental plaque [29]. Digestion of viral surface proteins by trypsin has also been reported [30–32].

Respiratory viral infection is a major cause of morbidity and mortality worldwide [33]. The most commonly detected viruses causing respiratory infections are respiratory syncytial virus (RSV), rhinovirus (RV), and influenza virus [34]. For most respiratory viruses, clinically useful antiviral agents do not exist [33]. Cod trypsin in a specific formulation has high efficacy against the H1N1 and H3N2 influenza viruses both *in vitro* and on skin tissue without affecting mammalian cell viability [35, 36]. Also, cod trypsin is shown in this review to have high efficacy against the two enveloped viruses: HSV-1 and RSV *in vitro*. 

A common side effect of respiratory viral infection is increased susceptibility to bacterial coinfection [33]. The most common coinfecting bacteria are known to form biofilm under certain conditions that may have serious health consequences, especially in young children [37]. An early preventive treatment of upper respiratory tract viral infection with a mild and nontoxic antipathogenic agent like cod trypsin may be effective in reducing the RSV and influenza viral load and possibly prevent bacterial coinfection. In the USA alone, about 100,000 hospitalizations of infants and young children are associated with RSV infections. Today, no comprehensive drugs or vaccinations are available for the fight against RSV [38]. 

Presently, infection by HSV-1 is incurable and the virus becomes latent in neural ganglia of the human body. Cold sores (blisters) are the most common herpes disease symptoms, but the virus can also cause severe infections in eyes and encephalitis [39]. Infections by HSV-1 can be controlled by antiviral drugs, usually acyclovir- and penciclovir-derived drugs that can reduce the viral load in the body. However, resistant HSV-1 strains have emerged against these drugs [40, 41]. 

## 2. Basic Research on Cod Trypsin

The Atlantic cod is known to produce numerous trypsin isozymes [7, 8, 42]. Several of these have been isolated from their native source, with trypsin I being the most predominant one. It also has the highest catalytic efficiency and is by far the best characterized of the trypsin isozymes [7, 8, 20]. The complementary DNAs of two trypsin isozymes (I and X) [8, 42], in addition to a novel trypsin termed trypsin Y [43], have been isolated from a cod pyloric ceca cDNA library. The pyloric cecum serves the role of a digestive organ in the Atlantic cod.

In general, trypsins from the Atlantic cod, Atlantic salmon (*Salmon salar*), and other fish adapted to cold environments differ somewhat from their mammalian analogues in that they have higher catalytic efficiencies, especially at low temperatures [7, 8, 22, 44, 45]. These enzymes are also more sensitive to inactivation by heat, low pH, and autolysis than their mesophilic analogues [7, 8, 46]. These traits and the fact that the cold-adapted enzymes function properly at low temperatures have stimulated interest in their commercial use, as they are generally better suited for enzymatic processes than their mesophilic counterparts [14, 47–49].

Numerous authors [10, 44, 45, 50, 51] have pointed out that the mechanisms of cold adaptation are more complex than previously anticipated, as enzymes seem to have adapted to cold in different ways [52]. One feature that appears to be common to cold-adapted enzymes is high molecular flexibility compared with their mesophilic analogues [53]. Recent reports show a strong relationship between high flexibility, high catalytic activity, and low thermal stability of cold-adapted enzymes [52, 54, 55]. 

The precursor form of the Atlantic cod trypsin I has been previously produced in an *E. coli *expression system and an active recombinant trypsin I was yielded through cleavage of a purified fusion protein [20]. Two cold-adapted protein expression systems *Escherichia coli* and *Pseudoalteromonas haloplanktis *were recently tested for the expression of recombinant cod trypsin I [21]. The results show that the *P. haloplanktis* system is better suited for the expression of the recombinant trypsin I than the *E. coli* system in terms of activity obtained and the amount of protein expressed.

Production of highly purified native Atlantic cod trypsin from pyloric ceca is not only environmentally friendly but also economically feasible. Therefore, cod offal is an important resource of trypsin. However, recombinant cod trypsin would be better suited to meet pharmaceutical standards and could provide a stable complimentary source of the enzyme. Active research and development are ongoing on the expression of recombinant cod trypsin in microorganisms. Also, site-directed mutagenesis has been used to improve production and stability of the recombinant cod trypsin [1, 21].

Expression of Atlantic cod trypsin I [20] and trypsin Y [56] were the first reports on the expression of active cold-adapted proteolytic enzymes from fish. The sensitivity of cold-adapted proteases to autolytic degradation, thermal inactivation, and molecular aggregation, even at temperatures as low as 18° to 25°C may explain the problems observed with their expression, activation, and purification [7, 8, 57, 58]. These problems may account for the limited number of publications in this area.

Most studies on the enzyme activity of cold-adapted fish trypsins have made use of small chromogenic substrates for activity measurements [7, 22, 59]. None of these studies show enzymatic activity towards proteins in their native conformation. In the past few years, research has been performed on the ability of Atlantic cod trypsin to degrade native proteins [2, 3].

**Figure 1:**
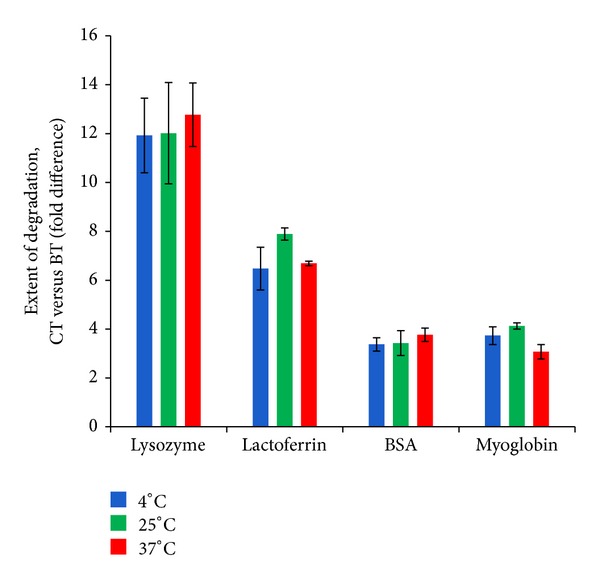
Degradation of different native proteins by cod trypsin compared to bovine trypsin. The columns show the fold difference in efficacy of cod trypsin (CT) over bovine trypsin (BT) in degrading the substrate proteins (lysozyme, lactoferrin, BSA, and myoglobin) in their native forms [2, 3]. The extent of degradation by cod trypsin is higher than that of bovine trypsin against all substrates tested. The enzyme digests were performed at 4°C for 72 hours, 25°C for 24 hours, and at 37°C for 3 hours. Error bars are standard errors of the mean based on at least triplicate experiments.

The superior efficacy of cod trypsin in degrading lysozyme, lactoferrin, BSA, and myoglobin in comparison to bovine trypsin is shown in Figure 1 [2, 3]. The degradation study was performed at three different temperatures over a period of 3–72 hours (at 4°C for 72 hours, at 25°C for 24 hours, and at 37°C for 3 hours). Degradation peptides were separated by reversed-phase chromatography and the extent of degradation was determined based on the resulting chromatograms. Figure 1 shows that the degradation is greater with cod trypsin compared to bovine trypsin for all temperatures tested. The proteins used as substrates are considered to be excellent model substrates as their structures, except for myoglobin, are adapted to the extracellular environment, and cod trypsin is topically applied when used for medical purposes. Furthermore, lysozyme, lactoferrin, BSA, and myoglobin have quite different structures, important for demonstrating the ability of cod trypsin to cleave various proteins in their native form.

 A significant difference in favor of cod trypsin over bovine trypsin is observed in the extent of degradation with all the substrates at the three temperatures tested (Figure 1). The fold difference ranges from about threefold (BSA and myoglobin) to about twelvefold (lysozyme). A change in temperature, from 4°C to 37°C, seems not to affect the difference in degradation between cod trypsin and bovine trypsin. The fold difference is about the same for each substrate at the three temperatures tested. The extent of degradation by cod trypsin compared to bovine trypsin is highest at 25°C (lactoferrin and myoglobin) or at 37°C (lysozyme and BSA). Previous studies on cod trypsin using small chromogenic substrates show as well the superior degradation ability of cod trypsin compared to its mesophilic bovine analogue [3, 8]. 

**Figure 2:**
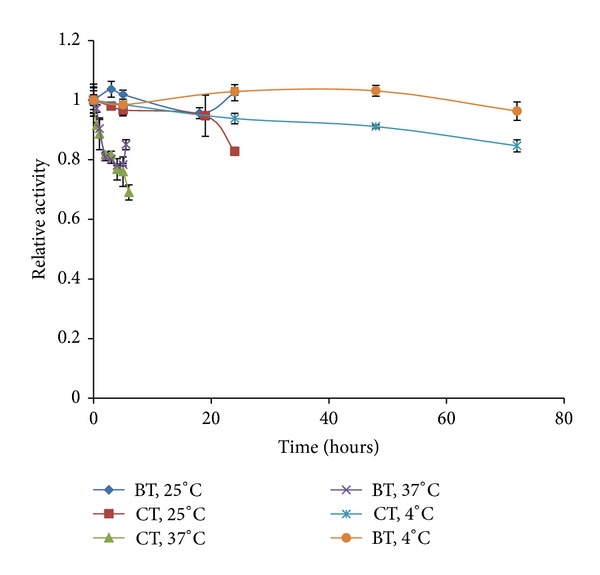
Stability of cod trypsin (CT) and bovine trypsin (BT) under the same conditions as those used for degradation of the native proteins lysozyme, lactoferrin, BSA, and myoglobin [2, 3]. The enzymes were incubated at 4°C, 25°C, or 37°C. Samples were taken at different time points and the activity towards the substrate CBZ-Gly-Pro-Arg-pNA was measured. The graph shows the activity of cod trypsin and bovine trypsin relative to their activity at the zero time point. Error bars are standard errors of the mean based on triplicate experiments.

The stability of cod trypsin and bovine trypsin was tested under the conditions used for cleaving the native proteins as seen in Figure 2 [2, 3]. The activity of bovine trypsin stayed almost the same after incubation for three days (72 hours) at 4°C and for one day at 25°C (24 hours). However, there was a fifteen percent decrease in the activity of cod trypsin under the same incubation conditions. After 6 hours of incubation at 37°C, there was a fifteen percent drop in the activity of bovine trypsin and thirty percent loss in the activity of cod trypsin. The fold difference in the extent of degradation between cod trypsin and bovine trypsin for each substrate was about the same at the three temperatures tested. The results indicate that changes in temperatures up to 37°C have minimal effect on the difference in the extent of degradation between cod trypsin and bovine trypsin.

 It is interesting to note the superior degradative ability of cod trypsin over bovine trypsin despite the lower thermal stability of the former enzyme (Figure 1). Surprisingly, the degradation potential of cod trypsin and bovine trypsin increases proportionally by increasing the incubation temperature from 4°C to 37°C. As a cold-adapted enzyme, cod trypsin has been postulated to have greater flexibility than its mesophilic analogues [53]. This quality could be the reason for the high degradation ability of cod trypsin towards large native proteins compared to bovine trypsin as increased flexibility may give rise to improved access of the enzyme to the substrate [45, 60]. Cod trypsin was shown to cleave all four substrates (lysozyme, lactoferrin, BSA, and myoglobin) at the same sites as bovine trypsin but at a much faster rate [3]. 

**Figure 3:**
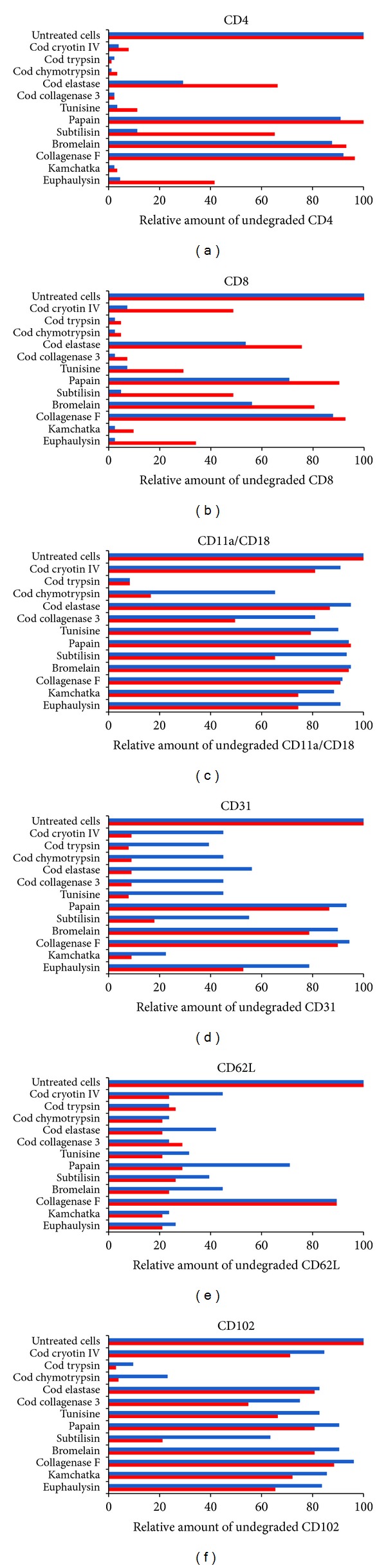
The ability of different proteases to cleave the surface receptors CD4 (a), CD8 (b), CD31 (c), CD11a/CD18 (d), CD102 (e), and CD62L (f) at the surface of freshly isolated murine T cells [27]. The proteases used are shown on the left side of the Figures (a)–(f). The proteases were used at a final concentration of 2 *μ*g/mL (blue bars) and 20 *μ*g/mL (red bars) for 2 h at 37°C. The bars represent the relative amount of undegraded surface receptors after digestion by the proteases tested, that is, the smaller the bars the higher the proteolytic efficacy. Each assay was carried out in duplicate or triplicate and the cells were T lymphocytes of murine and human origin. Each column represents average numbers. Standard deviation ranged from 0.01 to 0.1.

Research performed on 12 comparable proteases, including euphaulysin (protease from Antarctic krill), demonstrated the superior efficacy of cod trypsin and cod chymotrypsin in degrading various pathological proteins and cytokines [27] (Figures 3(a)–3(f)). The proteases tested were collagenase F, bromelain, subtilisin, papain, tunisine (tuna protease extract), and the following proteases from cold-adapted marine organisms: Antarctic krill euphaulysin, kamchatka (red king crab collagenase), Atlantic cod cryotin IV (cod protease extract), Atlantic cod collagenase, Atlantic cod elastase, Atlantic cod chymotrypsin, and Atlantic cod trypsin. Standard flow cytometry assay was used to measure the ability of the proteases to cleave cell surface receptors. In short, cells (2 × 10^6^) were incubated in the presence of various concentrations of protease (2–20 *μ*g/mL) for 2 h at 37°C. The enzyme was removed and quantification of various cell surface receptors was measured by staining the cells with specific fluorescent-labeled monoclonal antibodies. The fluorescence measured on individual cells by flow cytometry is directly related to the number of receptors at their surface. Each assay was carried out in duplicate or triplicate and the cells were T lymphocytes of murine and human origin. Cod trypsin and cod chymotrypsin were shown to be the most active enzymes against CD4, CD8, CD11a/CD18, CD31, CD62L, and CD102 at the surface of freshly isolated murine T cells (Figures 3(a)–3(f)) [27]. Very few enzymes, other than cod trypsin and cod chymotrypsin, were capable of cleaving integrin (Figure 3(c)). Trypsin was the most active enzyme in cleaving integrins CD11a/CD18 and CD102 at low enzyme doses (2 *μ*g/mL) (Figures 3(c) and 3(f)).

## 3. Antipathogenic Properties of Cod Trypsin *In  Vitro*

**Figure 4:**
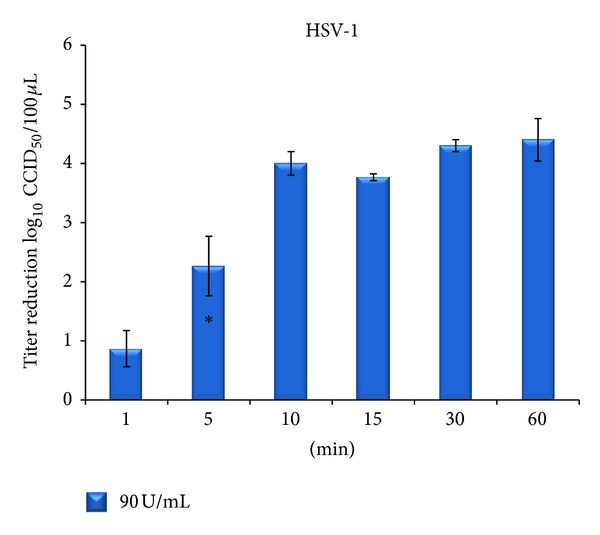
Reduction in HSV-1 titer after treatment with 90 U/mL final concentration of cod trypsin at different incubation times at 37°C [4]. Titer numbers were compared to a virus control in maintenance medium for each time point. Each column represents average number ± standard deviation of 3 independent experiments. No significant difference was seen in virus titer after treatment with blank solution when compared to virus control for each time point (data not shown). *The shortest incubation time causing significant reduction in titer when compared to the virus control (*P* < 0.01).

The ability of cod trypsin to reduce the viral titers of the enveloped viruses, HSV-1 and RSV [4], was analyzed as previous studies demonstrated high efficacy of cod trypsin against influenza viruses both *in vitro* and on skin [35, 36]. Time-dependent efficacies of cod trypsin against HSV-1 are shown in Figure 4. The columns represent the reduction of infectivity titer, log_10_ CCID_50_, of cod trypsin at a concentration of 90 U/mL after variable incubation times with HSV-1 before inoculation onto Vero cells. Virus mixed with MM or blank solution (data not shown) served as a control and was used for calculations of infectivity reduction. No difference was seen in viral titers of the blank treatment when compared to the MM virus control. The error bars represent the standard deviation of the mean for three independent experiments for each time point tested. After 5 min, cod trypsin at a concentration of 90 U/mL reduced the virus titer more than 100-fold, causing over 99% virus reduction (*P* < 0.01). After 10 min, the reduction was about 10 thousandfold with the same cod trypsin concentration, and further reduction was seen with increased incubation times. After 60 min of incubation, reduction of the HSV-1 titer was 4.4 log_10_.

**Figure 5:**
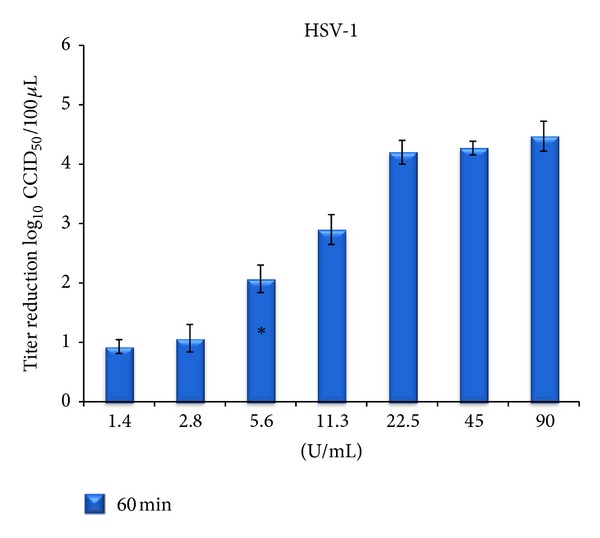
Reduction in HSV-1 titer following incubation with different final concentrations of cod trypsin after 60-minute incubation at 37°C [4]. Titer numbers were compared to a virus control in maintenance medium after 60 minutes. Each column represents the average number ± standard deviation of 3 independent experiments. No significant difference to the virus control was seen in virus titer after treatment with blank solution for 60 minutes (data not shown). *Lowest cod trypsin concentration causing significant reduction in titer when compared to the virus control (*P* < 0.01).

The efficacy of a lower concentration of cod trypsin was tested at a 60 min incubation time with HSV-1 (Figure 5). Cod trypsin at a concentration of 5.6 U/mL caused more than a 100-fold (*P* < 0.01) reduction in virus titer, while lower concentrations showed about 10-fold reduction. Cod trypsin at a concentration of 11.3 U/mL caused about 1000-fold reduction in HSV-1 titer, while higher concentrations, that is, 22.5, 45, and 90 U/mL all caused over 10 thousandfold reduction in virus titer after 60 min of incubation.

To further test the efficacy of cod trypsin against enveloped viruses, three different concentrations of cod trypsin at four time intervals were tested against RSV inoculated onto MA cells by a similar assay as that used for HSV-1. The results in Figure 5 show that after 1 min, both the 66.6 U/mL and 50 U/mL of cod trypsin showed about 10-fold RSV virus reduction. After 10 min of RSV incubation with 66.6 U/mL cod trypsin, a 1800-fold RSV virus reduction (3.3 log_10_) was seen. However, incubation of the virus with 50 U/mL and 25 U/mL cod trypsin caused a 2.5 and 1.8 log_10_ reduction in RSV, respectively. Increased incubation time showed further inactivation of the RSV. Thus, after 60 min over 100 thousandfold RSV viral titer reduction was seen after incubation with 50 U/mL cod trypsin and no CPE could be detected in the cell monolayer after this treatment. 

**Figure 6:**
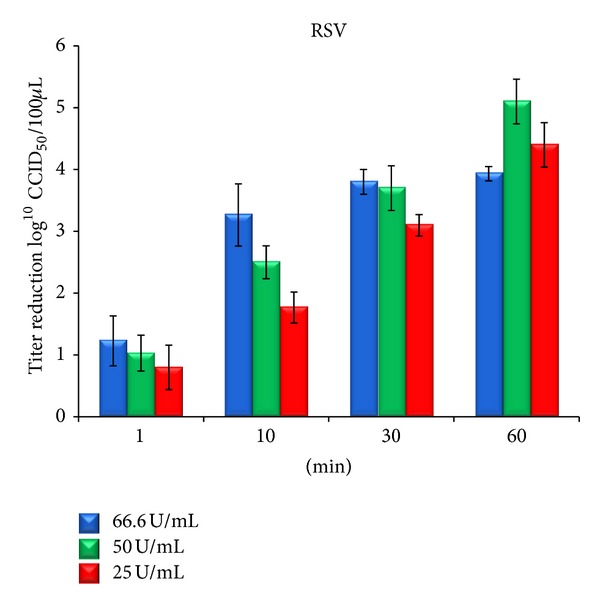
Reduction in RSV titer following incubation with different final concentrations and incubation time of cod trypsin at 35.5°C [4]. Titer numbers were compared to a virus control in maintenance medium for each time point. Each column represents the average number ± standard deviation of 3 independent experiments. No significant difference was seen in virus titer after treatment with blank solution when compared to virus control for each time point (data not shown). *Shortest incubation time and lowest cod trypsin concentration causing significant reduction in titer when compared to the virus control (*P* < 0.01).

The results in Figure 6 show that after 1 min, both the 66.6 U/mL and 50 U/mL of cod trypsin showed about 10-fold RSV virus reduction. After 10 min of RSV incubation with 66.6 U/mL cod trypsin, an 1800-fold RSV virus reduction (3.3 log_10_) was seen. However, incubation of the virus with 50 U/mL and 25 U/mL cod trypsin caused a 2.5 and 1.8 log_10_ reduction in RSV, respectively. Increased incubation time showed further inactivation of the RSV. Thus, after 60 min over 100 thousandfold RSV viral titer reduction was seen after incubation with 50 U/mL cod trypsin and no CPE could be detected in the cell monolayer after this treatment.

The higher activity of 50 U/mL cod trypsin compared to 66.6 U/mL at 60 min for RSV is due to detaching the cell effect of the 66.6 U/mL concentration sample to the MA cell monolayer. The first tenfold dilution of the 66.6 U/mL cod trypsin sample used in this assay, that is, 6.66 U/mL cod trypsin, causes detachment of MA cells from cell wells. Therefore, the titer could not be estimated more precisely than ≤1.5 Log_10_ CCID_50_, while 5.0 U/mL concentration of cod trypsin (first dilution of 50 U/mL sample) is well tolerated by the MA cells and no CPE was seen in that dilution. 

## 4. Upper Respiratory Tract Infections

Respiratory tract infections are caused by the synergistic and antagonistic interactions between upper respiratory tract viruses (respiratory syncytial virus (RSV), rhinovirus (RV), and influenza virus) and three predominant bacterial pathogens: *Streptococcus pneumoniae*, nontypable *Haemophilus influenzae *(NTHi), and *Moraxella catarrhalis *[37]. These bacteria are members of the commensal flora of the nasopharynx and can behave as opportunistic pathogens of the middle ear when conditions are optimal. A recent study on patients with respiratory virus infection found RSV to be more prevalent than RV and influenza viruses in children up to five years of age [61]. However, RV was found to be the most common infection identified in patients of all ages followed by RSV and influenza viruses.

Respiratory viruses promote bacterial adhesion to respiratory epithelial cells [62–64]. As adhesion is the first step toward colonization and infection, this viral priming has impact on disease. Treatment with cod trypsin at this stage to prevent adhesion could be important in preventing further progress of infection. The efficacy of cod trypsin in a specific formulation has been tested against RSV and influenza with positive effects. These are two out of the three main viruses known to cause upper respiratory tract infections. In addition, cod trypsin has been shown to prevent the adhesion of biofilm forming bacteria to mammalian cells *in vitro *(unpublished results).

## 5. Biofilm

Biofilms are an important factor in the pathogenesis of, for example, dental caries, urinary tract infections, and medical instrument colonization [65, 66]. In recent years, biofilm formation has been suggested to be important in the pathogenesis of diseases of the airway including sinusitis, bronchitis, and otitis media (OM middle ear infections) [37]. Methods are being developed to use enzymes for dispersal and elimination of biofilms from the middle ear space [37].

The Centers for Disease Control and Prevention (CDC, USA) estimates that biofilms account for two-thirds of the bacterial infections that physicians encounter. Such biofilms consist of clustered microorganisms where the cells adhere to each other on a surface [67]. The microbial cluster is embedded within a self-produced matrix of extracellular polymeric substance (EPS) [68, 69]. The role of bacterial biofilms in medicine is not entirely clear. The sessile life of microbes in biofilms has many advantages over the planktonic (free floating) lifestyle. It allows nutrients such as carbon, nitrogen, and phosphate to be concentrated in the sticky polymeric matrix surrounding the bacteria [67]. Microbes in biofilm are also shielded from harmful factors in their environment and are able to dissociate from the biofilm and colonize new areas.

The tendency of microbes in aquatic environments to lead a sessile lifestyle, forming biofilms, has been known since the 1940s. Biofilms are omnipresent in nature and it has been estimated that most prokaryotes on earth live that way [67]. Biofilms can also be formed in the tissues of patients such as in wounds or on medical devices like implants, where they can cause severe medical problems [67, 70]. Bacteria in biofilms are highly resistant to traditional antibiotic treatment [67, 71, 72].

Bacterial surface proteins contribute significantly to adhesion and several key proteins have been identified as being important for *staphylococcal* biofilm formation [67]. The microorganisms that are most frequently associated with medical devices are *Staphylococcus epidermis*, *Staphylococcus aureus* (SA), and *Pseudomonas aeruginosa *[67]. Biofilm formation can be thought as a virulence factor, a bacterial strategy that contributes to its ability to cause an infection [67, 73].

Biofilms are suggested to be a key factor in nonhealing wounds, and an explanation for the continuous inflammatory state of chronic wounds is lacking [74, 75]. Several studies support the theory that enzymes, on their own or as supplements to other methods, can be very effective in reducing or eradicating biofilms [76–78]. Proteases in general can be quite useful against biofilms and have been shown to have superior ability to degrade biofilms compared to other enzymes tested [72]. Preliminary data from our laboratory suggest that this is true for cod trypsin (unpublished results).

Cod trypsin is currently being tested clinically against methicillin-resistant *Staphylococcus aureus* (MRSA), which is one of the most common hospital-acquired bacterial infections [79]. These bacteria can form biofilms in wounds and become very resistant to traditional antibiotic treatment [71, 79]. Preliminary results from an *in vitro* study on the antipathogenic efficacy of cod trypsin against MRSA and SA performed in our laboratory indicate that cod trypsin can reduce adhesion of these bacteria to mammalian cells (unpublished results). After one hour of incubation with 20 U/mL of cod trypsin at 37°C, half of the SA had been removed from the cell culture and three-quarters of MRSA had been removed. These proportions were assessed based on control cell cultures that were infected with SA and MRSA but not treated with cod trypsin. Cod trypsin seems to work better against MRSA than wild-type SA indicating that the surface proteins of the drug-resistant *Staphylococcus aureus *mutant (MRSA) are easier to digest.

## 6. Wound Healing: A Clinical Study on Cod Trypsin Formulation

Enzymatic wound debridement prepares wounds for healing by removing nonviable tissue until the surrounding healthy tissue is exposed [80]. Debridement consequently plays a part in the treatment of wounds, especially chronic nonhealing or indolent wounds [81]. Debridement also inhibits production of inflammatory cytokines and reduces bacterial bioburden [81]. The debridement of necrotic tissue is mainly achieved in five principal ways: by autolysis, surgical intervention, mechanical methods, and biosurgery and enzymatic approaches. All have their place in therapy but the clinical need of the patient and the acceptability of treatment must be the main deciding factor. Mammalian trypsins have been experimented with since the early 1950s for wound management [82–84]. Today, bacterial collagenases are most commonly used for enzymatic wound debridement [80, 85, 86].

The cold-adapted characteristics of cod trypsin can be beneficial for use in wound debridement. Thermal inactivation of the cold-adapted cod trypsin, involving unfolding and autolysis, limits the lifespan of the enzyme in the wound bed, thereby minimizing the risk of harm to viable tissue. Trypsin loses its ability to degrade proteins at the normal pH of healthy skin (pH of about 5.5).

A clinical study was performed using a specific hydrogel formulation containing cod trypsin on pressure wounds at the Department of Pharmacy, Faculty of Medicine and Surgery at the University of Malta, Msida, Malta [5]. The clinical trial was performed on 32 patients with 50 pressure sores. The hydrogel containing cod trypsin (5 U/mL cod trypsins) was compared to other four conventional treatment products for highly severe pressure sores in the wound healing study. The study involved 50 wounds from St. Vincent de Paul Geriatric Residence (SVPR). These 50 wounds were divided into two equal groups: A and B. Group A was treated with the hydrogel containing cod trypsin while conventional treatment was used for Group B. 

The clinical efficacy of the products involved the management of pressure sores in these two groups was assessed for 25 weeks. Statistical analysis was carried out using Microsoft Excel and Biomedical Data Package. Health gain was being considered as a clinical cure in this study. The procedure followed for pressure sores was daily application of cod trypsin in a hydrogel formulation. The trypsin-hydrogel was allowed to dry and the wound was then covered with suitable dressing. In the control group, the wounds were cleaned and conventional treatments, Intrasite gel (Smith & Nephew Ltd.), Kaltostat dressing (Pharmacy only, ConvaTec Ltd.), Granuflex dressing (ConvaTec Ltd.), and Povidone Iodide 10% aqueous solution (Betadine, Purdue Products L.P.), continued to be used. 

**Figure 7:**
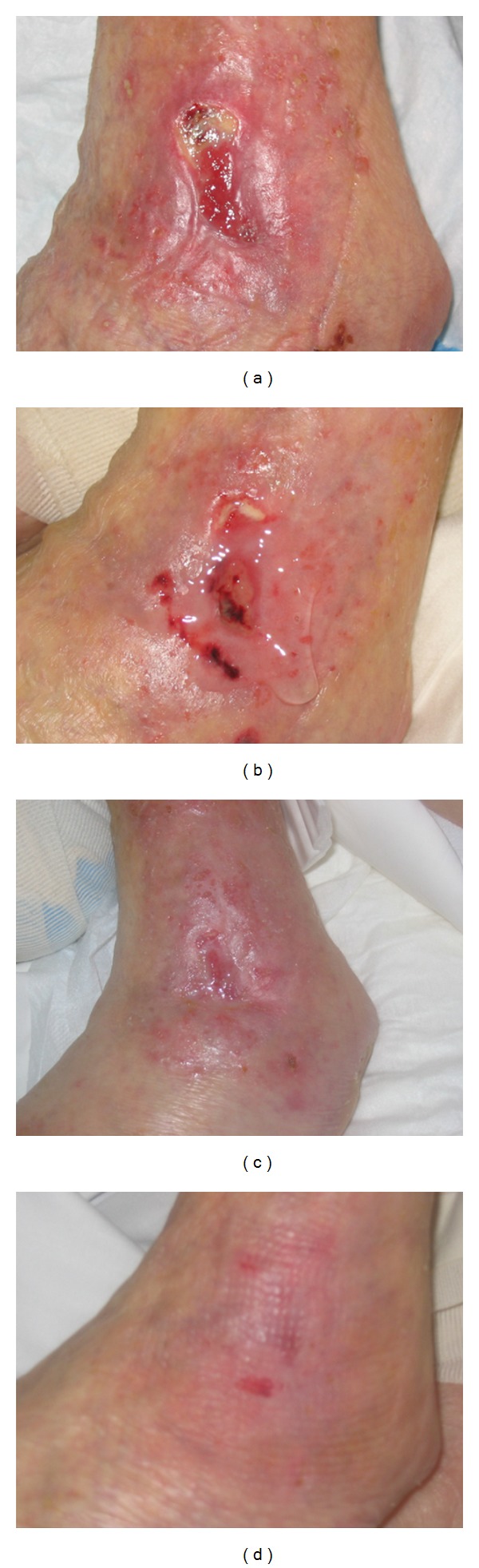
The photographs show the healing of a pressure sore when using a formulation containing cod trypsin [5]. The pressure sore on a left ankle before initiation of treatment with the cod trypsin hydrogel (5 U/mL) (a), after 5 weeks of treatment (b), after 9 weeks of treatment (c), and after 14 weeks of treatment (d) [5].

Figures 7(a)–7(d) show photographs of pressure ulcers healed by the use of cod trypsin in the specific hydrogel formulation. The mean duration of treatment for each patient in the study was 17 weeks. Group A mean duration was 15.84 weeks while that of Group B was 18.16 weeks. The results showed that the mean width of the pressure sores in Group A was significantly smaller than that of Group B in the final weeks of the study according to the Mann-Whitney test (*P* value ranging from 0.0221 to 0.0454). The same trend was seen with regard to the length of the pressure sores but the difference was not significant. The mean depth of the pressure sores in Group A was found to be significantly smaller than that of Group B from the beginning of the study (*P* value ranging from 0.0013 to 0.0386); hence the choice of wounds was biased from the beginning with regard to the depth of pressure sore. The colour of the wound appeared to be significantly better with the hydrogel containing cod trypsin treatment for several weeks (*P* value ranging from 0.0180 to 0.0411).

Cod trypsin in the specific hydrogel formulation was found to be of superior efficacy for wound healing compared to the conventional treatments. This was measured by a decrease in size (length, width, depth, and volume) of the wounds, improvement of color of the wound, and number of patients healed. Cod trypsin and its healing properties in combination with the moist wound environment from the hydrogel played a role in the healing effect. There are limitations to many of the available wound healing studies including those on enzymatic debridement like this study. However, the results are of importance to the wound care specialists for reasoning the best treatment of a particular wound type. The cod trypsin hydrogel was found to be useful for certain pressure sores that were difficult to manage and where no improvement was achieved with other products previously used. The cod trypsin hydrogel was found to be easy to use and no side effects were encountered as a result of its use.

## 7. Safety Considerations

Cod trypsin is thought to promote wound healing by facilitating debridement. *In vitro* studies show that cod trypsin is an effective antipathogenic agent against certain viruses and bacteria. These properties of cod trypsin are thought to be largely based on its effectiveness in degrading proteins [3]. Protein cleavage by cod trypsin in wounds is analogous to cleavage of food proteins by trypsin in the digestive system of humans. The enzyme efficiently cleaves food proteins without harming the epithelial cells lining the intestines. Topical formulations containing cod trypsin have been used with high safety in natural skin products for over a decade. Cod trypsin has been shown to be safe for human use [5, 87]. Pathogens originating from marine organisms, like the Atlantic cod, are less likely to be transmitted to humans than those from livestock, the source of bovine trypsin [1]. Today, no human pathogens from marine sources have been reported in the literature. Furthermore, the Food and Drug Administration (FDA) classifies trypsin as a GRAS (generally regarded as safe) substance.

## 8. Conclusion

The results presented in this paper demonstrate that cod trypsin has high efficacy in degrading native proteins and it shows antipathogenic efficacy against HSV-1 and RSV *in vitro*. Furthermore, cod trypsin was previously shown to have efficacy against influenza viruses. The results on the antipathogenic properties of cod trypsin are important because RSV and influenza are known to be two of the three most predominant pathogenic viruses in upper respiratory tract infections.

The question of how one enzyme like cod trypsin can have efficacy in so many different areas like wound healing and as an antipathogenic agent lies at least in part in its ability to cleave pathogenic proteins in an efficient manner. Cod trypsin has been shown to digest proteins on the surface of mouse cells in the absence of membrane trafficking. Thus, cod trypsin may disarm certain viruses and bacteria by cleaving their surface proteins. Our results also indicate that cod trypsin may be used to disintegrate and digest bacterial surface proteins important for biofilm formation or integrity. Cod trypsin could have value in the future as a natural, mild, and nontoxic antipathogenic agent. Future research will focus on clinical studies using cod trypsin as an antipathogenic agent against respiratory tract infections and biofilms. The natural form of cod trypsin will remain an important source for the enzyme, but the development of large-scale production of recombinant cod trypsin will be continued. Results from the clinical study presented in this review show that the specific formulation containing cod trypsin was preferred for wound healing over traditional methods.

## Abbreviations

HSV-1: Herpes simplex virus type 1
RSV: Respiratory syncytial virus
RV: Rhinovirus
CPE: Cytopathic effect
FBS: Fetal bovine serum
MM: Maintenance medium
CCID_50_: Cell culture infectious dose with a 50% end point
CT: Cod trypsin
BT: Bovine trypsin
SA: *St* *ap* *hy* *lo* *co* *cc* *us* *aureus*
MRSA: Methicillin-resistant *Staphylococcus* *aureus*.
